# First-Line Managers’ Experiences of Leading a Multicultural Staff Group in Nursing Homes and the Implementation of Language Development Initiatives: A Qualitative Study

**DOI:** 10.1177/10436596251330601

**Published:** 2025-03-31

**Authors:** Elisabet Eriksson, Cecilia Arving, Katarina Hjelm

**Affiliations:** 1 Uppsala University, Sweden; 2 University of Gävle, Sweden

**Keywords:** leadership, aged, cultural diversity, language development, nurse manager

## Abstract

**Introduction:**

Foreign-born care workers with limited native language proficiency have become a challenge for first-line managers (FLMs). The study explores FLMs’ experiences of their role of leading a multicultural and multilingual staff group in nursing homes and implementing language development initiatives.

**Method:**

This is an explorative descriptive study. Four focus-group discussions were held with 12 participants. Data analysis was performed in accordance with the method described for focus-group discussions.

**Results:**

Three categories with six subcategories were generated: Challenging to adapt leadership to multicultural environment; Challenging and stimulating to work with language development; and Challenging to bridge gaps when conditions change.

**Discussion:**

FLMs’ role to implement different strategies and models to promote a favorable working environment, collegial relationships, and competence development is demanding. In conclusion, FLMs need sufficient time, resources, and support to develop competence in transcultural nursing leadership to deliver culturally congruent care and implement language development initiatives in multicultural teams.

## Introduction

Due to staff shortages within elderly care, foreign-born care workers with limited native language proficiency often choose to work at nursing homes (NHs), even if they have no previous experience of or education in elderly care provision (Krohne et al., 2019). This presents a challenge for first-line managers (FLMs). Research has shown that FLMs are essential to ensuring a favorable working environment, collegial relationships and competence development at the ward level. However, it has been emphasized that more research is needed on the integration of strategies and on FLMs’ role in implementing language development initiatives (Kamau et al., 2022; Teixeira et al., 2024). Thus, the present study will explore FLMs’ experiences of leading a multicultural and multilingual staff group in NHs as well as of implementing language development initiatives, the goal being to fill some of the knowledge gaps in this field.

Nursing leadership and management are performed at the first-line, middle, and executive/senior levels within health care organizations (Engle et al., 2017). Thus, a wide range of competencies is required to meet all of the expectations associated with the FLM role (Asplund et al., 2022; Gunawan et al., 2018). The role of FLMs at NHs is often described as complex, and it is carried out in an environment marked by changes in older patients’ requirements as well as staffing (Asplund et al., 2022; Jordal et al., 2022; Wastesson et al., 2022). In Sweden, foreign-born individuals comprise about 20% of a population of 10 million (Statistics Sweden, 2022). The definition of a foreign-born care worker is an individual who comes from a population group whose culture, ethnicity, language and religion are different from those of the majority population (Adebayo et al., 2020; Eriksson et al., 2023). In Sweden, approximately 50% of the care staff or nursing aides working in elderly care are foreign-born, and approximately 30% have no training/education (Eriksson et al., 2023; The Swedish Association of Local Authorities and Regions, 2024). However, in one review, NH residents reported being satisfied with the care provided by the multicultural staff group (Adebayo et al., 2020). In addition, employers preferred employing foreign-born care workers for whom respecting elders was a norm (Tibajev et al., 2022), as this made them better care workers (Adebayo et al., 2020). However, foreign-born care workers may experience challenges in adjusting to the workplace culture as well as face discrimination and communication difficulties (Adebayo et al., 2020; Eriksson & Hjelm, 2022a, 2022b; Eriksson et al., 2023b). Addressing these challenges requires integration strategies, which can be grouped into three domains: intraorganizational, sociocultural, and professional development (Kamau et al., 2022). In the first domain, intraorganizational strategies were tailored specifically to the characteristics and needs of a particular organization. Strategies included policies that facilitated cultural and linguistic diversity in the workforce and collegial support. In the second domain, sociocultural language and communication were developed through strategies such as organization-supported language learning. The third domain included professional development strategies that improved competence and professional development. The essential role of FLMs in ensuring favorable integration of their multicultural and multilingual NH staff has been emphasized in several studies (Chen et al., 2020; Debesay et al., 2022; Kamau et al., 2022; Munkejord, 2019; Teixeira et al., 2024). However, to our knowledge, the topic of FLMs’ experiences of their role in implementing language development initiatives among multicultural NH staff has not been addressed, and thus, more research is needed. Accordingly, the present study’s objective is to explore FLMs’ experiences of their role in leading a multicultural and multilingual NH staff group and implementing language development initiatives.

## Method

### Design

A qualitative explorative study design, with data collected through focus-group interviews (FGIs), was used (Patton, 2015). FGIs were chosen because group interaction improves participants’ ability to express their experiences; they also encourage participants to disclose attitudes they might not willingly reveal in one-on-one situations (Krueger & Casey, 2015).

### Setting

FLMs worked at NHs in a municipality in central Sweden with approximately 242,100 inhabitants, 23% of whom were born abroad (Statistics Sweden, 2022). In 2020, the Senior Citizens Board instructed the municipality to strengthen its work with language development. Since then, several initiatives to support language development have been implemented through a strategy entitled “Language development workplaces.” In 2023, 66 language assistants were trained (1-day training), four of whom were further trained as language advocates (4-day training). The language assistants have been assigned to support their colleges in communication and create a safe and accommodating atmosphere at NHs.

### Sampling and Procedure

Purposive sampling was used to include FLMs from different areas in the municipality. All FLMs (*n* = 23) at public NHs in the municipality received an email with study information from process managers at the municipality; the FLMs were encouraged to reply by email if they were interested in participating. Twelve agreed to participate. Most had attended some language development training. Two were registered nurses, and the other participants had previous work experience in social work, elderly care, marketing, and communication. For participant characteristics, see Table 1.

**Table 1.** Characteristics of the Study Population, FLMs.

Variable	FLMs *N* = 12
Age (years)^1^	44.5 (27–71)
Gender (*n*)	
Female	8
Male	4
Level of education (*n*)	
Education at university level ≥ 2 years^2^	11
Current working conditions	
Full time	11
Part time	1
Working as FLM (years)^1^	10 (1–23)
Number of years working at the current nursing home (years)^1^	2 (0.5–5)
Country of birth (*n*)	
Sweden	8
Bosnia and Herzegovina	1
Iran	1
Cuba	1
Sri Lanka	1
Languages FLMs reported understanding (*n*)	
Swedish	12
English	11
French	2
Bosnian	1
German	1
Greek	1
Persian	1
Serbo-Croatian	1
Spanish	1
Tamil	1
Attended any language training in the role as FLM	
Yes	9
No	3

*Note.* FLM = first-line manager.

1 Values are Median (Range). ^2^One missing value.

### Data Collection

Four FGIs, characterized by lively interaction between participants (2–4/group), were conducted between February and March 2023. The interviews, which lasted for 82 to 90 min, were recorded and led by a moderator, a nurse (first author) and an assistant moderator (second author). The FGIs were held in secluded conference rooms that were familiar to the participants (Krueger & Casey, 2015). The small-group dynamic did not compromise data richness, as the fluent discussions and lively interaction resulted in 169 A4 pages of transcripts. The results rely more on the interaction and involvement of the participants in each group than on the number of participants (Krueger & Casey, 2015).

An interview guide was developed by the research team. It was based on a literature review and previous studies covering participants’ sociodemographic data, experiences of a multicultural context at NHs, implementation of language development initiatives and leadership. The first session was used as a pilot test (Krueger & Casey, 2015), after which some minor changes were made to the wording and sequencing of questions.

### Data Analysis

Data analysis was performed in accordance with the method described for FGIs (Krueger & Casey, 2015). Collection and analysis of data proceeded simultaneously, until no new information emerged (Krueger & Casey, 2015). The FGIs were transcribed verbatim, generating data rich in content. The transcripts were exported to Open Code (ICT Services and System Development and Division of Epidemiology and GlobalHealth, 2013) for data organization. The first author began the analysis by reading through the transcripts to get a sense of the whole. Text strings related to the study objective were identified and marked as meaning units in the transcripts. These were coded as close to the text as possible. Groups of codes sharing common attributes were organized into subcategories and then clustered to form categories. The co-authors also read the transcripts, checked and discussed the content of the codes in relation to the subcategories and categories until consensus was reached (Patton, 2015). The Consolidated Criteria for Reporting Qualitative Research (COREQ) were followed (Tong et al., 2007).

### Ethical Considerations

The study was approved by the Swedish Ethical Review Authority (Reg. no. *2020–03636 and 2022-01084-02*). Written informed consent was obtained from all participants prior to the FGI. Participation was voluntary, and participants were told they could withdraw from the study at any time. Anonymity between the focus-group participants could not be guaranteed, which they were informed of. They were instructed to share information only during the interviews. Confidentiality was ensured by pseudonymizing the data, which were handled/stored according to regulations.

## Results

**Figure 1.**
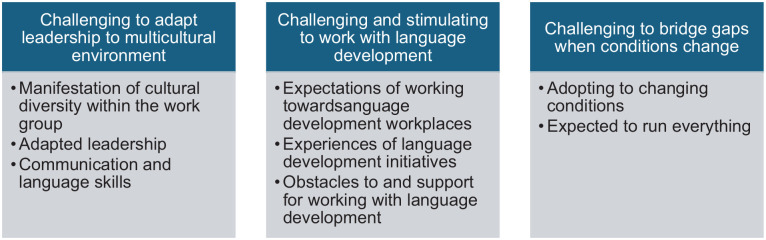
Overview of the Three Categories With Subsequent Subcategories.

Three categories emerged from the data: Challenging to adapt leadership to multicultural environment; Challenging and stimulating to work with language development; and Challenging to bridge gaps when conditions change (Figure 1). Illustrative quotes from the FGIs are provided; numbers in parentheses represent each FGI.

### Challenging to Adapt Leadership to Multicultural Environment

FLMs discussed many expressions of cultural diversity within their workforce as well as how they had adapted their leadership style and communication depending on whether they were addressing Swedish or foreign-born staff.

#### Manifestation of Cultural Diversity Within the Work Group

FLMs reported that 50% to 90% of their employees were born abroad and represented many cultures and countries:

“Many have an immigrant background and . . . well, actually more than 90%.” . . . “We have people from Bangladesh, Afghanistan, Iran, Poland, China. So, like the whole world.” (FGI 1, Respondent 1)

Only one participant reported having more Swedish than foreign-born staff. Regarding gender, most of the staff were women, although some reported mixed groups. The age of the staff varied between 30 and 50 years. Staff members’ work experience varied from having no previous experience to more than 20 years. Foreign-born staff mainly had experience from work in Sweden. Accordingly, the FLMs felt that the multicultural representation influenced the socialization process and communication among staff. FLMs described some advantages of having a multicultural workforce, such as appreciation on the part of foreign-born residents:

Then I have to say that it’s good to have people who speak other languages because more and more people are coming to the residence who weren’t born in Sweden, then having a multicultural staff is a plus. (FGI 2, Respondent 1)

However, FLMs also reported that having many different cultural groups among the staff was demanding. They described misunderstandings between Swedish and foreign-born staff that they had to resolve. Furthermore, meal situations could be perceived as strange when foreign-born staff served residents food without asking how to prepare it:

Something simple like serving Swedish food can go wrong, the condiments can be totally wrong. Like serving porridge with ketchup. Things like that can happen, have happened. (FGI 2, Respondent 1)

All FLMs reported that staff were obliged to speak Swedish at the workplace, yet they found it difficult when staff spoke their mother tongue. They said that, in those situations, others were excluded and there was a risk of different ethnicity-based groups forming in the workforce. Related to this, FLMs described the occurrence of threats and violence against staff and themselves, some tied to criminal networks whose members feared other criminal networks. However, the employees did not dare file a police report. FLMs at some NHs described how a culture of silence had become the norm and that they were not receiving any support in handling these situations. Some Swedish FLMs reported lacking knowledge about different ethnic cultures. They said it was impossible to know everything about all cultures, and they therefore expressed themselves very cautiously:

I think it’s hard when there are so many different cultures and I don’t know. I don’t know everything about all cultures, so I don’t know if I’ll just happen to say something to you that you find offensive, or vice versa. Today everybody’s scared to death of being called a racist. (FGI 1, respondent 1)

#### Adapted Leadership

Management of a multicultural workforce was reported to be different from managing Swedish staff. Some FLMs found it easier to lead foreign-born staff because they had more respect for them as managers. However, this great respect for them was also problematic, because the foreign-born staff were unlikely to ask questions. Therefore, the FLMs were unsure about whether foreign-born staff had understood information, for example, during a staff meeting. To encourage silent staff to speak, the FLMs had organized the working groups into smaller groups representing different ethnic groups. In addition, when encouraging staff to follow routines, FLMs reported that foreign-born staff were loyal to them as individuals rather than to the laws and regulations. Another cultural difference was described by female FLMs when male staff had difficulties accepting them as managers. Moreover, the FLMs discussed how they adapted their leadership and felt that foreign-born staff wanted them to use a more authoritative leadership style, while the Swedish staff desired a more consultative style:

They [foreign-born staff] want me to give strict instructions and they want to follow them. But I can’t relate like that to my . . . those in the working group who are ethnic Swedes. There it’s more like a conversation. (FGI 1, Respondent 2)

#### Communication and Language Skills

Some reported that Swedish language skills among staff constituted a minor problem, while others described limited Swedish language skills as a major problem. Issues described were complaints from Swedish employees, quarrels between staff, and complaints from relatives. Some pointed out that language problems should not be underestimated and that there should be higher standards for Swedish language skills among staff. Misunderstandings resulted in things not turning out as expected; foreign-born staff did not follow routines, gave the wrong medication, and did not make notes in residents’ records. When something went wrong, the FLMs had to put more energy into figuring out why:

There are two aspects here if someone is uncertain about the language, compared to someone who knows the language. Then you can be a bit tougher, otherwise you have to put more energy into figuring out why something went wrong. Don’t you want to do it or don’t you understand? (FGI 3, Respondent 3)

The FLMs felt they were very clear in their communication with foreign-born staff and adapted their language:

If I’m going to write a risk assessment because a user is acting out, then I’ll write “run.” I don’t write “back out slowly and avoid eye contact.” I write what I need to say, “get out of there.” (FGI 3, Respondent 2)

### Challenging and Stimulating to Work With Language Development

All FLMs felt the municipality expected them to work with language development, although they expressed varying levels of interest in leading such initiatives. Moreover, they reported mixed experiences of and lack of support in implementing different language initiatives among their staff.

#### Expectations of Working Language Development Workplaces

According to the FLMs, they were expected to work with language development at the NHs. These expectations were based on survey responses from relatives, the language requirements for hiring NH staff, and the municipal administration. At the same time, some FLMs were hesitant about conducting language training at the workplace. While some FLMs found it interesting to work to improve foreign-born staff members’ Swedish language skills, others were not interested. They said they had no formal education in this area and questioned why the municipality thought work with language development workplaces was a suitable task for FLMs:

In the best of worlds, I wouldn’t have to deal with this myself. Instead . . . If the municipality initiates something then they also need to ensure it gets done . . . with their own . . . what are they called, project-based employees. They need to take charge of it // instead of what it’s like now: You’re supposed to do this and you’re supposed to start it up too. (FGI 1, Respondent 3)

#### Experiences of Language Development Initiatives

FLNs reported having had experience with many different language initiatives, such as shorter digital courses, language cafés at the workplace, and employees being allowed to learn Swedish during working hours. Other initiatives from the municipal Care and Welfare Department involved training language assistants and language advocates. However, many FLMs found this demanding because the different roles were unclear both to them and the persons given these titles. Furthermore, language assistants needed a great deal of support from the FLMs:

Well, I haven’t found anyone who wants to be a language assistant for . . . I also haven’t been able to say what a language assistant actually does. (FGI 2, Respondent 2)

### Obstacles to and Support for Working With Language Development

One challenge associated with work toward language development workplaces was the different needs expressed by staff. Some of them were illiterate, and a few had an academic education:

Because it’s a communication thing, so to speak. Maybe they write . . . aren’t good at Swedish, but they don’t dare send an email, like you say. And if they would we could also . . . the communication would be better. (FGI 1, Respondent 1)

Moreover, FLMs reported that foreign-born staff needed to be motivated and that it was difficult for them to encourage employees to participate in language initiatives. Furthermore, they did not know how to document their employees’ improvements in language skills. They were also unsure about what initiatives had the best effects. The main hindrances reported were lack of knowledge, time, and resources:

I can set up some kind of . . . Well, different kinds of Swedish courses. That would be a lot of fun, but then I need to be given the prerequisites, the time and the resources. (FGI 2, respondent 3)

Regarding support, the FLMs thought any language initiatives should be managed at a more central level in the municipal organization. They wanted more coordinated activities and consensus regarding the role of language assistants and advocates. They wanted the municipality to employ language teachers who could teach their staff at the NHs. Others expressed the need to have access to cultural anthropologists and pedagogical materials on language development. The question remained of how language development should be pursued on wards where no one is a native speaker of Swedish.

### Challenging to Bridge Gaps When Conditions Change

FLMs reported that they must adapt to a more multicultural society and that they were sometimes forced to hire people who had no interest in elderly care. Moreover, they were expected to lead many different projects despite having limited resources.

#### Adapting to Changing Conditions

FLMs reported that the prerequisites had changed during recent years, from them mainly leading a Swedish workforce at NHs to leading a multicultural workforce. The reasons they gave were that few Swedes seek vacant positions at NHs, while foreign-born individuals apply for employment to improve their chances of being granted a residence permit. They described how they had to adapt and felt compelled to hire people who had been trained, through national support programs, to be assistant nurses and who had been promised employment. According to the FLMs, some of them were not interested in working with people in elderly care, had limited Swedish language skills, and stayed in their positions to meet work experience requirements for obtaining a residence permit:

They stay pretty long because it has to . . . They often need another job in order to advance. And today it’s not that easy to get a job either. So they stay. A lot of them stay for many years, I must say, after they’re done. (FGI 1, Respondent 2)

I think that, like we were saying about it, maybe all of them shouldn’t . . . Shouldn’t work there, you know, the incentive is to get a residence permit rather than wanting to work with people. (FGI 1, Respondent 3)

#### Expected to Run Everything

The FLMs felt pressure to lead many different projects initiated by the municipality. Moreover, they were expected to find staff willing to take on the responsibility of being representatives for these projects. FLMs could have up to 16 different projects involving representatives and they felt the municipality did not prioritize among the projects. Leading many projects was also challenging, as they could not be experts on everything. Working toward language development workplaces was just one of the many projects placed on the managers' table, and they had limited time and resources to follow up on the projects:

Because it . . . . In our part of it, I feel it just dies somewhere. They tell us about it first and then we’re supposed to take over. But we don’t have enough resources, time, knowledge to manage all these projects. (FGI 1, Respondent 3)

## Discussion

The present study is unique in that it explores FLMs’ experiences of their role in leading a multicultural and multilingual staff group in NHs and implementing language development initiatives. The main findings showed that leading a multicultural and multilingual staff group involved demands and challenges that required FLMs to adapt their communication and leadership style.

The FLMs reported having adapted their leadership style when addressing foreign-born staff, as these staff members expected their FLM to be authoritative. FLMs’ role has been described as complex (Gunawan et al., 2018; Jordal et al., 2022), and the present study adds that FLMs must constantly navigate between different leadership styles, consultative and authoritative, which puts extra demands on their role. This is in accordance with the Theory of Culture Care Diversity and Universality, also known as the Culture Care Theory (CCT) (Leininger, 1988), which suggests that leaders must adapt to cross-cultural patterns of health care beliefs and practices, inter- and intra-cultural communication, and transcultural ethics. Moreover, FLMs reported facing threats to themselves and staff from ethnicity-based groups within the workforce. To enhance communication and avoid ethnicity-related conflicts, FLMs had reorganized the staff into smaller cross-cultural and multilingual working groups. FLMs play a crucial role in creating an environment that enables positive cross-cultural encounters among staff (Chen et al., 2020; Leininger, 1988; Munkejord, 2019). FLMs must work within three domains—intraorganizational, sociocultural, and professional development—if they are to support successful integration of a multicultural and linguistically diverse elderly care staff group (Kamau et al., 2022).

Most NH staff were foreign-born, confirming that the context for FLMs has changed and that they have difficulties employing staff with sufficient Swedish language skills and work experience from elderly care. The shortages of skilled health professionals are expected to increase, especially in upper-middle-income countries like Sweden (Liu et al., 2017). For this reason, the recruitment difficulties FLMs face require prompt attention. National efforts have been made to increase opportunities for foreign-born people to work as assistant nurses (National Board of Health and Welfare, n.d.). Although these efforts are greatly needed and basic knowledge of the Swedish language is required for employment at NHs, the FLMs felt forced to employ newly trained nursing assistants with limited Swedish language skills. FLMs described this as the reason why misunderstandings sometimes occurred that risked patient safety. Our findings confirm results from previous studies showing that FLMs valued bilingual staff who could assist in language translation for residents in elderly care (Chen et al., 2020; Weston, 1966; Xiao et al., 2018). Moreover, FLMs reported that bilingual staff provided peer support during introduction of newly employed foreign-born staff. In 2022, The Swedish Government (2022) commissioned the National Board of Health and Welfare to develop support for facilitating language assessment among care workers employed by elderly care providers within the social services. Creating an inclusive organizational culture may take time, and FLMs need support during this process. Surprisingly, the FLMs reported a lack of support from their leader in managing these difficult situations. The results indicate that FLMs need increased support from human resources. Moreover, they should be offered coaching and tailored training programs to lead multicultural teams as well as to develop competence in transcultural nursing leadership (Teixeira et al., 2023), which emanates from The Sunrise Enabler by Leininger and could be a useful tool in learning about culture care and modes of care decisions and actions (McFarland & Wehbe-Alamah, 2019). Transcultural nursing leadership has recently been defined as (Teixeira et al., 2023) leading/guiding the delivery of culturally congruent care that addresses health disparities, and as building highly inclusive and productive nursing teams in an increasingly diverse world. It refers to a culturally sensitive transformational journey of adapting behaviors and processes to the cultural needs of staff and patients as well as breaking with conventional paradigms. The leader needs to guide the staff to consider, first, what caring decisions and actions should be preserved or maintained, second, what should be negotiated or accommodated and, as a last resort, what should be restructured and/or repatterned (McFarland & Wehbe-Alamah, 2019). It is important that municipal leadership understand that managing a multicultural workforce puts extra demands on the FLM role and that situations can occur that need specific and prompt attention. This is further supported by a recent scoping review (Teixeira et al., 2024) that pointed out the importance of nurse managers being supportive, culturally competent, and effective communicators. The review also showed that having a transformational leadership style is particularly beneficial, but concluded that more research is needed. Kamau et al. (2022) concluded that strategies for improving professional development, such as language training and certifications within the staff group, could be seen as part of FLMs’ remit. However, some FLMs discussed whether language training should be carried out at NHs and whether it should be included in their role. The reasons for their hesitancy were that they lacked formal skills in teaching Swedish, sufficient resources, and time. Moreover, they felt these initiatives should be managed at a more central level in the municipal organization. However, some FLMs were enthusiastic about implementing language development initiatives.

One limitation of FGIs is the risk that participants will influence each other’s opinions; however, managers are used to expressing their own opinions. In our study, the groups were composed of their area managers, thus individuals accustomed to discussing work issues with one another (Krueger & Casey, 2015). The sample size could be considered limited; however, redundancy level was reached in the data analysis (Patton, 2015). Because the data were carefully collected, analyzed, and described, the results are transferable to contexts with similar characteristics (Patton, 2015). Future studies are needed to determine which interventions aimed at improving communication skills among foreign-born NH staff are most effective and feasible.

## Conclusion

In accordance with Leininger’s Theory of Culture Care Diversity and Universality, FLMs role to implement different strategies and models to promote a favorable working environment, collegial relationships, and competence development is demanding. FLMs need sufficient time, resources, and support to develop competence in transcultural nursing leadership to deliver culturally congruent care and implement language development initiatives in multicultural teams. More research exploring the impact of transcultural nursing leadership on multicultural work is needed and such research is lacking in elderly care.
